# Manufacturing Thermoset Lattice Core Sandwich Panel via Frontal Polymerization

**DOI:** 10.1021/acsapm.5c04911

**Published:** 2026-03-27

**Authors:** Maxwell Condell, Ryan Ruiz, Erin Poyer, Aaron Vigil, John Arthur Stewart, Xiang Zhang

**Affiliations:** Department of Mechanical Engineering, 4416 University of Wyoming, 1000 E. University Avenue, Laramie, Wyoming 82071, United States

**Keywords:** lattice core sandwich panel, frontal polymerization, thermoset, dicyclopentadiene, additive manufacturing

## Abstract

Frontal polymerization (FP) is a promising polymer curing technique leveraging a self-propagating exothermic polymerization reaction front to rapidly and fully cure monomers into thermoset polymers upon initiation. Prior studies have demonstrated the feasibility of using this propagating reaction front in a direct-ink writing (DIW) setting for both free-form and layer-by-layer printing of polymer and composite structures. In this study, FP-based DIW is adopted to develop a manufacturing technique for thermoset lattice core sandwich panels (LCSPs) that eliminates the need for additional bonding steps between the lattice core and face sheets. Different types of lattice cores are first printed by using FP-based DIW, where an IR camera is used to monitor the front behavior and adjust the front velocity in real time to improve printing accuracy. Face sheets are subsequently fabricated and bonded to the lattice core by immersing the lattice core into a reservoir of ink and initiating FP, which eliminates the need for bonding typically seen in LCSP manufacturing. The fabricated panels are examined using an optical microscope, and compressive tests are conducted by using parts fabricated by fused deposition modeling (FDM) as a reference. The printed parts show reasonable dimensional accuracy and full bonding between the face sheet and lattice core without visible air bubbles or voids, to the precision of the microscope. The DCPD LCSPs have a much higher ultimate strength compared to that of their FDM counterparts.

## Graphical abstract

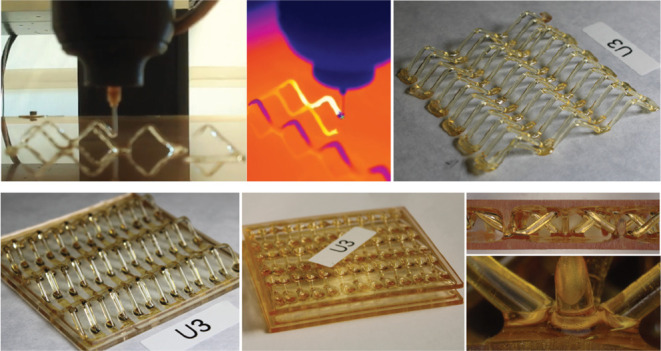

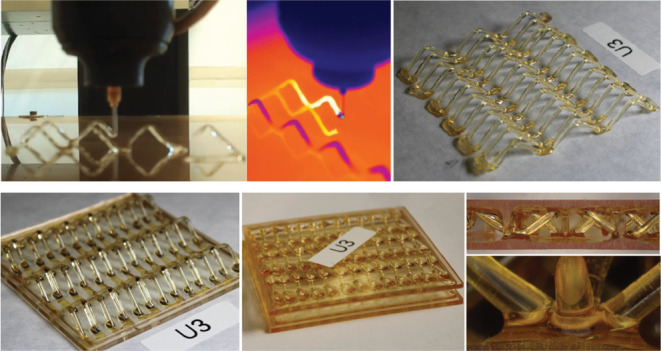

## 1 Introduction

Lattice core sandwich panels (LCSPs), typically made of a periodic lattice core and two face sheets, are desirable structures used in a wide range of engineering applications, including aerospace and automotive engineering, due to their high strength and stiffness relative to their weight. They also provide ample design space when fully explored by design processes^1^ and achieved by using different lattice core geometries, materials, and architectures,^2^ enabling extraordinary single,^3^ multiple,^4^ or spatially graded functionalities.^5^

When it comes to the manufacturing of LCSPs, especially polymer or polymer composite LCSPs, several challenges remain despite significant recent advancements. The first challenge comes from the geometric complexity of the lattice core. Different types of lattice geometry have been used for making polymer or polymer composite LCSPs, including tetrahedral, pyramidal, octet, and Kagome types (e.g., see a detailed review in ref 6). Hierarchical lattice cores introduce fine lattice architecture into the strut members of the lattice cores,^7,8^ or hybrid lattice cores combining struts and plates lattice members,^9^ are also emerging. Various fabrication techniques have been developed to manufacture lattice cores with low to moderate geometric complexities, including hot press molding,^10^ weaving,^11^ interlocking,^12^ and laser cutting.^13^ Recent advancements in additive manufacturing (AM) allow the fabrication of geometrically complex lattice cores in a single process, presenting great potential for fabricating LCSPs. For example, fused deposition modeling (FDM)^14,15^ and direct-ink writing techniques can directly extrude single-filament thermoplastic polymer lattices with discrete or continuous fiber reinforcement.^16,17^

While these thermoplastic polymers or polymer composites made by AM have shown great potential, 3D printing a lattice core with the improved mechanical properties of thermoset polymers is a second challenge we are currently facing. The improved mechanical properties of thermoset polymers over thermoplastic polymers make them more desirable in some critical applications.^18^ Both commercial- and research-driven strategies for the additive manufacturing of thermoset polymers employ a variety of techniques, including vat photopolymerization^19^ and DIW-based in situ curing by photocuring,^20–22^ microwave,^23^ radio frequency,^24^ or Joule^25^ heating. However, these techniques may lead to very thin printing layers due to limited light penetration, especially with fillers; require constant energy input during printing; or may require a second postprinting thermal curing process.^26–28^

An emerging curing strategy, frontal polymerization (FP), provides an alternative way for manufacturing thermoset LCSPs while addressing some of the aforementioned challenges. FP relies on a localized, exothermic polymerization reaction that provides enough energy to the surrounding monomer to self-propagate a polymerization front upon initiation.^29–31^ The self-propagating nature of FP reactions means that only a small initial heat source is required to initiate the reaction, after which the reaction front will self-sustain and polymerize a large quantity of FP-capable monomer. Recent experimental and computational studies have empowered a series of FP-based applications related to thermoset polymer and polymer composite manufacturing.^32–38^ By integrating FP with a DIW printing platform, it is possible to rapidly and efficiently print free-standing^39^ and layered^40^ structures. Additionally, different reinforcements can be mixed into the monomer ink to allow printing with composites.^41–44^

This work proposes a new LCSP manufacturing technique that leverages FP to address the previously mentioned challenges, as well as a third challenge for many fabrication methods: the need to bond the lattice core and the face sheets in a separate step. The polymer we are using is dicyclopentadiene (DCPD), but this method could be applied to other types of thermosetting resins that support FP, such as epoxies,^44–47^ urethanes,^48,49^ and acrylates,^50^ with (i.e., for composites) or without (i.e., for polymers) fillers.^41,42,44^ The process starts with the preparation of a DCPD-based FP-capable monomer ink. Next, a custom-built DIW printer setup with an infrared (IR) feedback system is used to print a series of lattice cores. The lattice cores are then placed into a face sheet mold and filled with neat resin with a depth equal to the thickness of the desired face sheet. This setup is then moved to an oven to initiate frontal polymerization of the ink in the reservoir to quickly cure the face sheet by FP without an additional bonding process. This process is repeated to form the face sheet on the other side, delivering thermoset polymer LCSPs. The printed parts are examined using a microscope, and the mechanical properties of the lattice core sandwich panels are studied and compared with those made by FDM using compressive tests.

The remainder of this article is organized as follows: Section 2 describes the developed method of lattice panel manufacturing using FP, followed by a discussion of the results based on this method in Section 3, including challenges faced and compression testing. Finally, Section 4 provides a summary of this work and proposes some future work to investigate.

## 2 Materials and Methods

The lattice panels consist of three components: a printed lattice core and two layered face sheets. All components are fabricated using the same FP-capable DCPD-based polymer ink. The ink is used in a custom DIW printer to print three different types of lattice core structures: a unidirectional triangular lattice (Figure 1b,c), a bidirectional triangular lattice (Figure 1d,e), and a pyramidal lattice (Figure 1f,g). These lattices are composed of the same unit triangle shown in Figure 1a. The completed lattices are then placed in molds with more DCPD ink to create the face sheets via FP and complete the lattice panels. While spacing in the bidirectional and pyramidal lattices is based on the full width of the unit triangle, the rows of the unidirectional lattice are spaced one-quarter of the length of the unit triangle apart. Each bidirectional lattice we used comprised four unit triangles, each 24 mm in length, creating 96 × 96 mm lattices. The unidirectional lattice is of the same size, which consists of 8 rows of lattice, and each row consists of four unit triangles. As the pyramidal lattice required alternating rows of triangles to be offset by half of a length, the pyramidal lattices were 102 × 102 mm^2^ in size.

**Figure 1:**
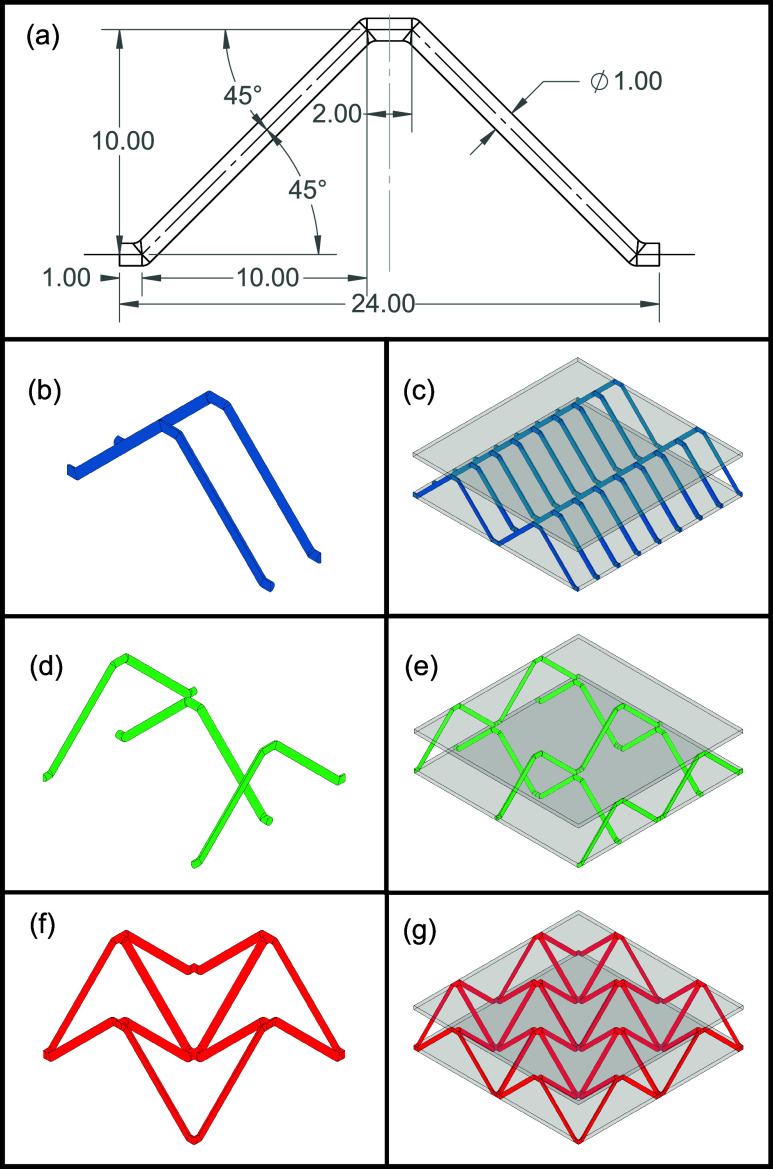
Illustration of three different types of LCSPs considered: (a) base triangle unit used in the lattice core, where the length unit is in mm; a unidirectional LCSP with the unit cell (b) and the full panel (c); a bidirectional LCSP with the unit cell (d) and the full panel (e); and a pyramidal LCSP with the unit cell (f) and the full panel (g).

### 2.1 Ink Preparation

Before the lattice may be printed, a batch of DCPD ink must be prepared using the same chemical composition as in our previous work.^40,51,52^ The first step in preparing the DCPD ink is to prepare the DCPD, which is a solid at room temperature, to be utilized in a liquid form. This is done by melting the DCPD in an environmental chamber set to 50 °C. The melted DCPD is then combined with 5-ethylidene-2-norbornene (ENB) in a ratio of 0.05257 g of ENB for every gram of DCPD (about 5% ENB by weight) to allow the DCPD to stay as a liquid at room temperature. This preparation of the DCPD/ENB mixture may be performed separately from the preparation of the DCPD ink.

When mixing a batch of DCPD ink, weighing Grubbs Catalyst 2 (GC2) in a glass test tube is started according to the quantity laid out in Table 1. In an argon-rich environment, the phenylcyclohexane (PCH) is added to the catalyst. The argon-rich environment helps maintain the purity and efficacy of the PCH through multiple DCPD ink batches, as the PCH may get contaminated by the environment. We then seal this mixture with Parafilm and sonicate it in a bath sonicator for 15 min. The sonication process may occur outside of the argon-rich environment, though the PCH source bottle should be resealed to prevent contamination before removing it from the argon environment. While waiting for the sonication process to finish, the appropriate amount of the previously prepared DCPD/ENB mixture may be measured out. When the sonication of the GC2 and PCH is finished, the test tube is returned to the argon-rich environment to add the tributyl phosphate (TBP), which is another contamination-sensitive chemical. The argon-rich environment is no longer necessary once the TBP source bottle is resealed. The contents of the test tube are then added to the DCPD/ENB mixture. A quick swirl or stir is necessary to help the two mixtures combine and form a resultant pink mixture. This mixture is then vacuum-filtered and prepared for usage or storage. It is important to note that the actual amount of ink produced is about 11% greater than the nominal batch size, which refers to an approximation of the amount of the DCPD/ENB mixture required. For example, the nominal 180 mL batch presented in Table 1 produced about 200 mL of ink. A video of this ink preparation process is provided in the Supporting Information.

**Table 1:** Quantities of Different Chemicals to be Mixed with 180 mL of the DCPD/ENB Mixture

chemical name	quantity
GC2	120 mg
PCH	6 mL
TBP	38.28 μL
DCPD/ENB mixture (95:5)	186.42 g

To store the DCPD ink before use, we divided it into sealed containers and placed them in the freezer at −30 °C. The ink can be stored in the printing barrel to facilitate easy transfer into the printer. As bulk polymerization will occur in the DCPD ink and cause the ink to gel to a state where it can no longer be easily poured or extruded, freezing the stored ink extends the amount of time the ink can be stored to about 2 weeks.

### 2.2 Lattice Printing

The custom printer used to print the lattice cores is built upon a commercially available AGS1000 Direct-Drive Gantry (AeroTech, LLC), as shown in Figure 2a, and consists of two coupled *X*-axis controllers, a *Y*-axis controller, two independent *Z*-axis controllers, and a linear actuator to control extrusion. The gantry is set on a custom-built table to support the complete printer. A heated bed is attached to the table, consisting of 3D-printed polylactic acid (PLA) plastic components, a sheet of 1/2-in. plywood, a heating pad (Omegalux SRMU101111 from OMEGA Engineering), and a borosilicate glass sheet as the printing surface. A PID controller (Platinum Series from OMEGA Engineering) is used to control the bed temperature. The bed also uses a set of screws to ensure that it is level.

**Figure 2:**
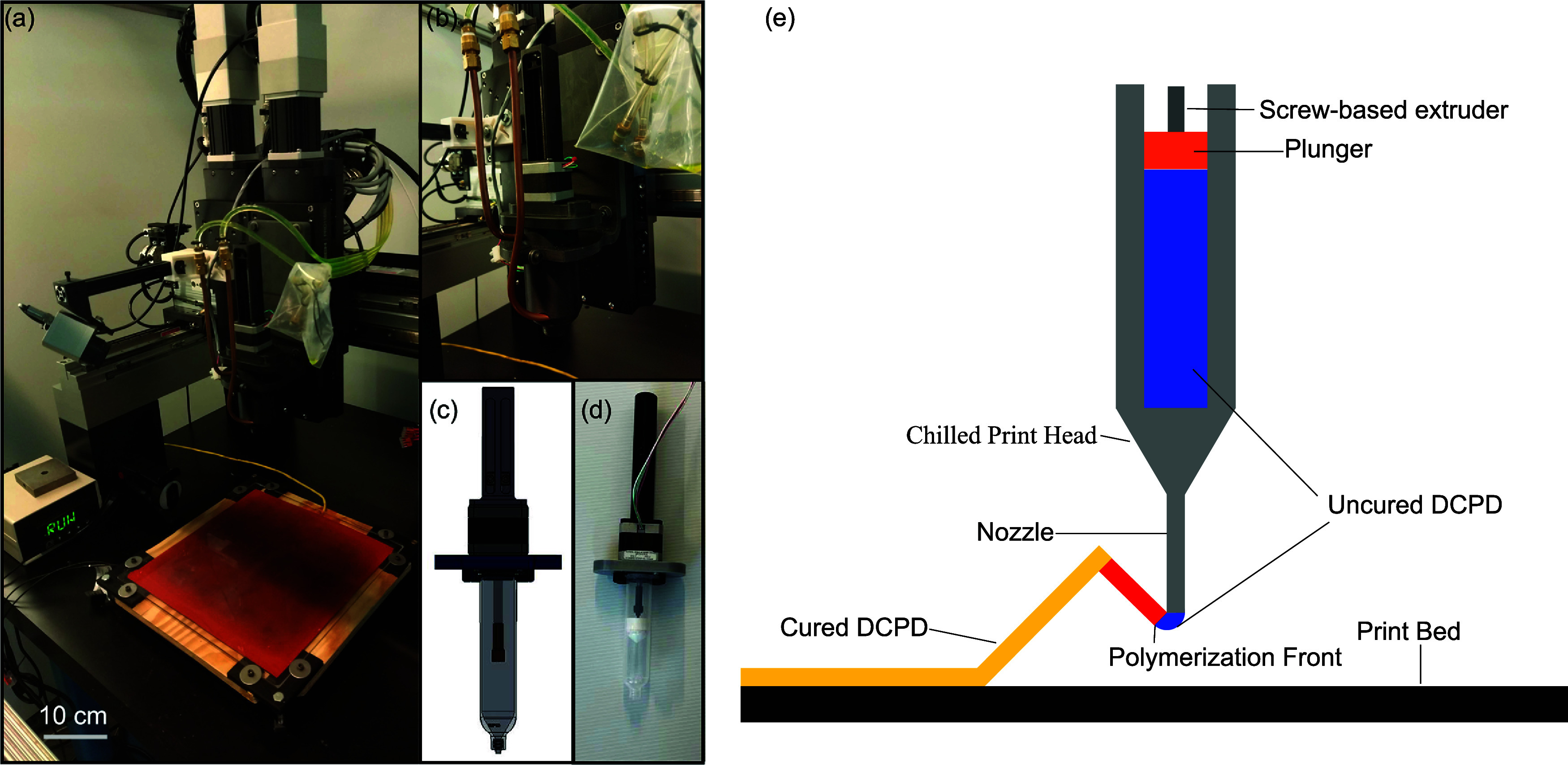
(a) Custom printer setup; (b) close-up of the chilled print head; (c) SolidWorks model of extrusion assembly; (d) completed extrusion assembly; (e) schematic illustration of the printing process, where the blue color of the DCPD in the print head and right after the nozzle indicates its cold temperature before FP, the red ink filament near the nozzle indicates its high temperature immediately after FP, and the yellow ink segment far away from the nozzle indicates its cooling down after FP.

Attached to the *Z*-axes are a custom-built print head and an infrared camera (PI 640i from Optris IR Sensing, LLC), both supported by printed PLA parts. The print head is made of 3D-printed ABS plastic and contains a coil of copper tubing for temperature control and an inner aluminum sleeve for heat transfer. The print head, as shown in Figure 2b, is designed to accommodate a 30 cm^3^ syringe (Nordson EFD). The print head is connected to a temperature-controlled bath (PolyScience AD07R-20) to control the temperature of the ink in the print head. The bath temperature is set at −5 °C, resulting in an ink temperature in the syringe of about 6 °C.

To perform free-form printing with the DCPD ink, the ink must be able to maintain the shape that the print head deposits until the polymerization front can pass through it. Simultaneously, it requires that the print nozzle stays far enough ahead of the front to avoid polymerizing the print head reservoir, as shown in Figure 2e. As the viscosity of the ink immediately after mixing is not sufficient to maintain free-form printing, an incubation process is performed to increase the viscosity through partial polymerization. Setting 20 mL of frozen ink in an environmental chamber at 30 °C and ambient humidity for about 52 min will achieve the desired consistency for printing in our current study, though times may vary based on the amount incubated and the degree of contamination in the PCH or TBP used.^39^ It is important to carefully monitor the ink as it approaches the desired viscosity, as a matter of minutes can be the difference between being too fluid to maintain cohesiveness and too viscous to extrude. The ink is incubated in a syringe to allow a quick transfer of the ink to the print head. This ink had a front velocity of about 1.0 mm/s in a test tube.

When the ink is incubated, the syringe is placed in the prechilled print head, and a 12.7 mm-long 15-gauge tip (i.e., 1.36 mm inner diameter and 1.65 mm outer diameter, Nordson EFD) is attached to use as a dispensing nozzle. The chilled print head helps to achieve two main goals: preventing continued bulk polymerization of the ink and preventing the polymerization from reaching past the nozzle into the syringe reservoir. While the chilled print head helps to mitigate the effect of the front being faster than the print head, further controls are necessary to help maintain an ideal nozzle lead distance over the front. As such, a C++ control loop, detailed in ref,^53^ was implemented to coordinate and adjust the speed of the gantry’s AeroBasic print code with temperature data from the IR camera. Figure 3 shows feedback from the C++ code and video captured by the IR camera and a separate webcam during a print. The monitoring feedback in the top left of the image reads out a maximum and minimum recorded temperature, although it is range-limited between −20 and 125 °C. Tests using a wider temperature range on the IR camera indicate that the front temperature peaks at around 240 °C, but using the range-limited reading, the system can estimate the front location. The amount of time it takes to print a lattice depends on the lattice and how much material is used for each lattice. For the lattices presented, the pyramidal lattice and the unidirectional lattice each took about 30 min to print, while the bidirectional lattice took 15 min. A video of the lattice printing process, showing the views from the optical camera and the IR camera, as well as real-time information such as print speed, the distance between the nozzle and the front, etc., is provided in the Supporting Information.

**Figure 3:**
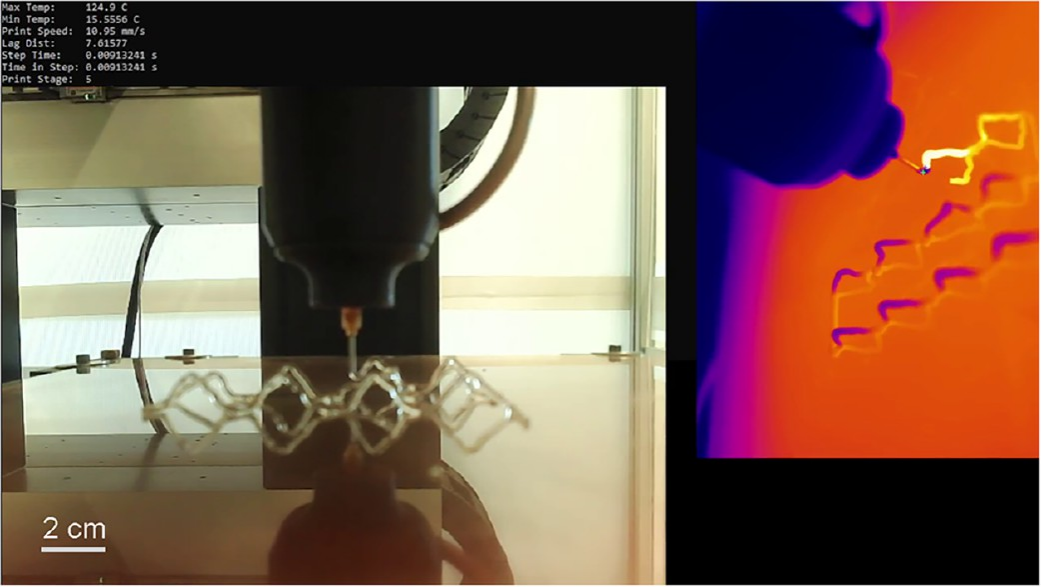
Print monitoring setup with program feedback: an optical camera view on the left and an IR camera view on the right.

### 2.3 Fabrication of the Lattice Core Sandwich Panel

A complete unidirectional lattice core is shown in Figure 4a. The bonding between the lattice core and the face sheets is achieved automatically by putting the printed lattice core into the DCPD reservoir before FP. The panel with a face sheet on one side is shown in Figure 4b, and this process is repeated to fabricate the other face sheet and deliver a complete panel as in Figure 4c.

**Figure 4:**
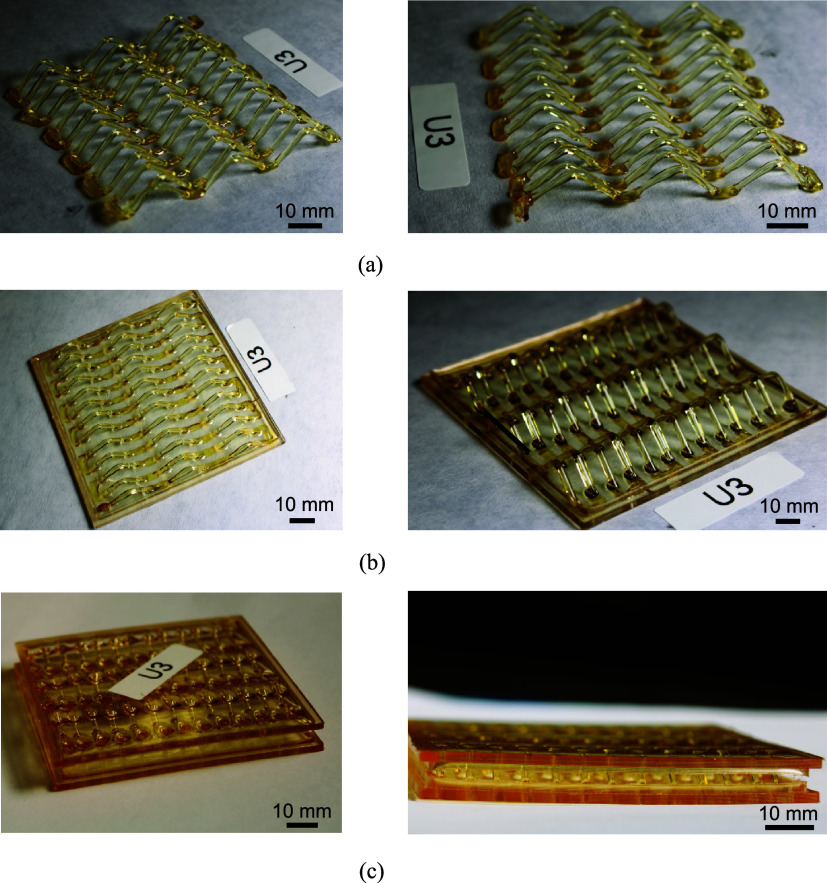
Illustration of the panel fabrication process: (a) printed unidirectional lattice core; (b) lattice core with face sheet on one side; (c) complete LCSP.

While FP of the face sheet can be initiated by a point heat source for energy efficiency, such as using either a soldering iron or a resistive heating wire, the fast reaction in an open container, especially at a low initial degree of cure (i.e., either the neat DCPD or the printing ink) with low viscosity, will cause surface patterns or excessive residual deformation.^54^ Additionally, accessing the resin by a soldering iron when fabricating the second face sheet is challenging, and a resistive heating wire requires additional devices and procedures.

To balance the time and energy needed for fabricating the face sheet, we developed a procedure by incubating the lattice core with the neat resin for 7 min at 70 °C. The 70 °C is an optimal choice based on our trials because it takes 4–5 min for the front to initiate, by which the ink partially cures and the viscosity increases, and then multiple fronts will initiate and propagate without excessive surface pattern or residual deformation. The total fabrication time, excluding the ink preparation and waiting time between steps, is about 29 min for the unidirectional LCSP and 44 min for the bidirectional and pyramidal LCSPs.

The mold consists of a sheet of borosilicate glass and water-jet 6061 aluminum side walls secured to the glass with hot glue. Using a vacuum oven allows the polymerization vapors to be evacuated before pulling the complete face sheet out. The lattices are attached to the face sheet by placing the lattice in liquid ink before curing the face sheet. This embeds the lattice into the face sheet, avoiding extra bonding steps.

To provide baseline comparison data, LCSPs with the same lattice geometries were fabricated by using fused deposition modeling (FDM) on a Bambu Lab X1 Carbon printer. eSUN PLA+ filament (1.75 mm diameter) was used for all prints. Each sandwich panel consisted of two face sheets and a lattice core. The face sheets were designed with nominal dimensions of 80 × 80 × 1.5 mm. The lattice cores were designed with a height of 8.5 mm and nominal strut diameters of 1.5 mm. All geometries were designed in SolidWorks to closely match the FP DCPD panels and were sliced using Bambu Studio. The LCSPs were printed in a vertical orientation without support structures. Eight lattice cores were printed simultaneously for testing. The total print time for the batch was 6 h and 1 min, excluding printer preparation time, with a total material usage of 218.1 g. After fabrication, dimensional measurements were taken. The face sheets had average measured dimensions of 79.69 × 79.84 mm with an average thickness of 1.56 mm. The lattice struts had an average measured diameter of 1.53 mm. It should be noted that it is beyond the scope of the current work to identify the best slicing and FDM printing settings for the three types of LCSPs, but by using established print settings and choosing an orientation to minimize the amount of support material used, one FDM panel of this size takes about 45 min to print, so it will take about 101 min (compared to 29 min for unidirectional LCSP and 44 min for bidirectional and pyramidal) to print a panel of the size of the bigger DCPD panel.

### 2.4 Part Quality Examination and Compressive Test

To understand the printed part accuracy and quality, the dimensions of each type of DCPD panel are measured at the four corners and the center. A regular camera (Canon Rebel T7) and a digital microscope (Keyence VHX-7000) are used to examine the lattice and its bonding with face sheets.

Flat-wise compression testing was performed as per the ASTM C365 ^55^ standard using the 858 mini Bionix II hydraulic load frame. The panel samples were crushed to 20–40% strain to ensure the lattice failed, so both the ultimate strength of the panel and the strength at 2% strain could be measured. The full DCPD panels are cut into smaller panels for testing to avoid the issues caused by warping in the larger panels. The cut DCPD samples are nominally 38.5 mm on each side. Smaller complete panels with three unit cells in each direction are also fabricated for testing. For the FDM panels, complete panels are used.

The specimens are also imaged during and after the compressive tests to understand the failure modes under compression. The stress–strain responses, together with the ultimate strength and stress at 2%, are compared between the DCPD panels and the FDM panels.

## 3 Results and Discussion

A fabricated LCSP is cut into smaller pieces for examination and testing. The examination focuses on the bonding between the lattice cores and the face sheet and the process-induced deformation of the fabricated parts, while the testing focuses on the out-of-plane compressive response. Some observed issues during the manufacturing process and possible mitigation methods are also discussed.

### 3.1 Lattice and Interfacial Bonding

Figure 5 shows, overall, the lattice struts have uniform thickness and smooth surface finish, with a nice elliptical cross section from the angle cut, suggesting good circularity of the struts. This high circularity of the printed filament is also observed in our previous work.^39^ The face sheet is slightly thicker around the bonding areas with the struts, which is caused by the surface tension when the lattice is immersed in the ink reservoir. Overall, the face sheet shows a reasonably uniform thickness in areas away from the bonding areas.

**Figure 5:**
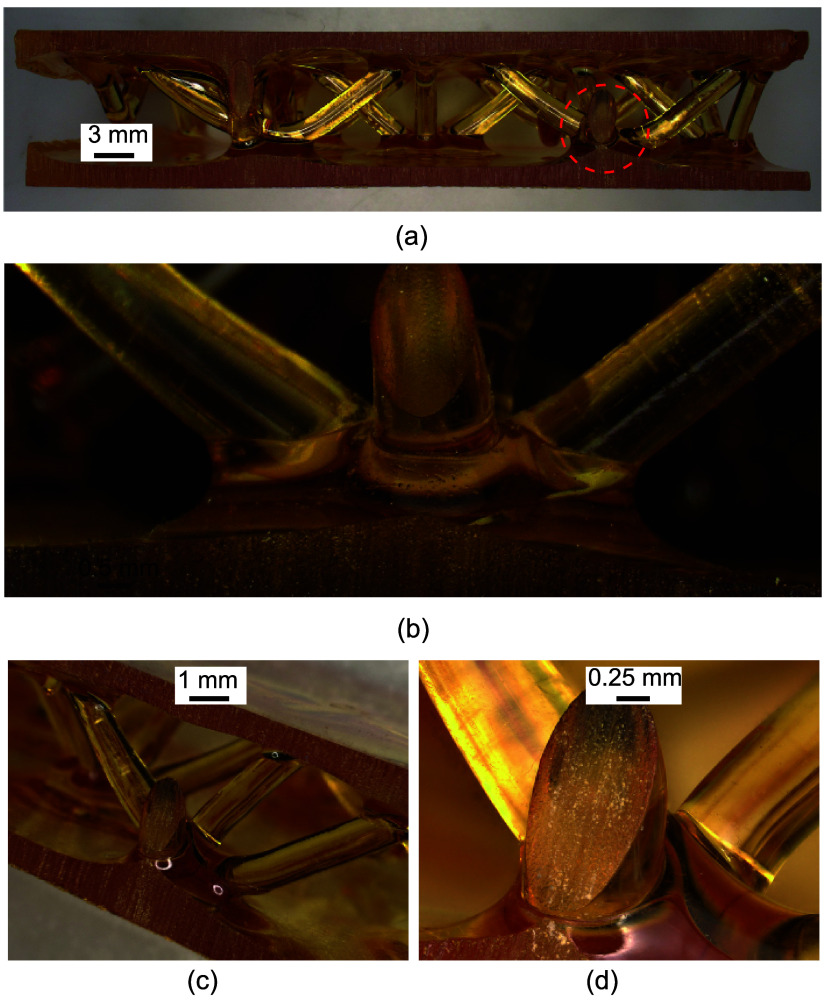
Details of the bonding between the lattice core and the face sheet: (a) side view of the cut sample, where the location in the red dashed circle is further magnified in parts (b–d). It should be noted that the bright circle in (c) is not a void or air bubble, but rather an artifact caused by lighting.

The bonding area between the struts and the face sheet is magnified in Figure 5b,c. While a visible interface is seen between the struts and the face sheet, the bonding appears to be free of defects or voids. Overall, the interface caused by curing a new monomer on previously cured polymer is expected to be weaker, which is typical and widely explored in AM parts, and pullout tests are needed to precisely quantify the bonding between the struts and the face sheet. Since most potential applications of these panels likely involve compressive loading, we therefore do not pursue the pullout tests in the current study.

### 3.2 Potential Issues and Mitigation

Figure 6 shows a few of the many failed prints and some of the causes. The ink’s viscosity significantly impacts how well it prints, not only for FP-based printing^39^ but also for DIW-based printing in general.^56,57^ The failure in Figure 6a is seen as a necking or breaking filament. This could be caused by the low viscosity of the ink due to insufficient incubation or a low extrusion rate, causing the extruded filament to shrink before curing.

**Figure 6:**
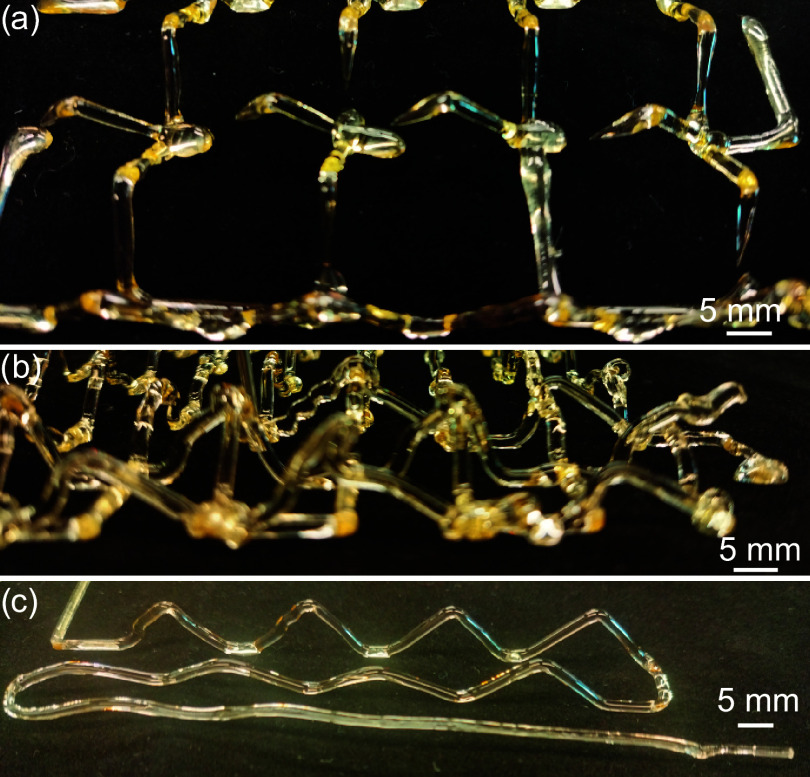
Illustration of failed lattice prints, where issues include: (a) low extrusion rate or low ink viscosity, (b) excessive lead given by the print head, and (c) excessive partial curing resulting in an unprintable gel, which may occur during the print.

The failure in Figure 6b is the significant deformation of the filament, which is typically the result of the print head being far enough ahead of the polymerization front to allow the extruded ink to sag before being cured. In our previous study, an empirical value of the maximum allowed distance between the nozzle and the front is identified.^40^ The inherent self-regulative behavior (i.e., to match the nozzle velocity) of the front driven by the interaction between the filament temperature gradient and cure kinetics is helpful for maintaining the desired distance between the front and nozzle. We used a preliminary computerized feedback control system by tracking the location of the front and the nozzle, as shown in Figure 3, which can be further improved with more sophisticated control algorithms and front tracking.

Figure 6c shows a print featuring a straight section of filament, deviating significantly from the desired lattice shape. This is because of the bulk polymerization happening in the syringe, which makes the ink viscous and hard to extrude. The nozzle is therefore applying an overly large extrusion force on the filament and dragging the filament into a stretched and straight shape. This issue can be mitigated by limiting the wait time between mounting the syringe and the printing process, as well as by limiting the ink temperature to a lower value.

In addition to the issues with printing high-quality lattices, the face sheets for the panels have some challenges in their fabrication. When curing a face sheet without lattice cores, we can obtain a very flat face sheet, as in Figure 7a. However, warping of the face sheet is often induced when creating the second sheet to complete the panel, which shows a different amount of warping depending on the lattice types as shown in Figure 7b–d. Other factors that may affect the amount of warping induced include the temperature of the oven used to initiate the FP and the time left in the oven after polymerization. To fully understand the warping process and identify potential mitigation plans by optimizing the processing parameters, thermo-chemo-mechanical modeling could be adopted^52^ rather than trial-and-error, especially when we scale it up to larger dimensions. The warping in the current study was not significant enough to affect the testing procedure and was mostly negated by the testing preload.

**Figure 7:**
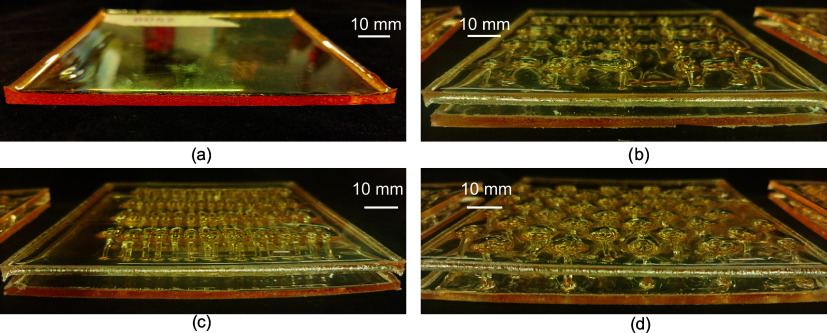
Notable issue in manufacturing: panel warping. Warping typically does not occur when creating the first face sheet (a) but occurs when creating the second face sheet and is inconsistent between panels. This is demonstrated with the bidirectional lattice (b), the unidirectional lattice (c), and the pyramidal lattice (d).

### 3.3 Compressive Testing Results

The uncut DCPD samples (three unit cells in each direction) and FDM samples are nominally 80 mm on each side. Three panels per lattice are taken to measure the thickness at the four corners and the middle of the panel, with the thickness of the panel being 9.38 ± 0.47, 9.03 ± 0.87, and 10.91 ± 0.99 mm, respectively, for the unidirectional, bidirectional, and pyramidal lattice types. This indicates that the unidirectional lattice panel has the best dimensional accuracy, while the pyramidal panel has the worst. It should be noted that the FDM panels have a much better dimensional accuracy and consistency between prints. Specimens were loaded at 0.5 mm/min.

Figure 8 and Table 2 present the flat-wise compression test data, showing the results from FDM panels (FDM-F), a full FP panel (DCPD-F), and cut FP panels (DCPD-C), grouped by lattice geometry. Corresponding ultimate strength and stress at 2% strain are also summarized in Table 2.

**Table 2:** Stress Data from Compression Testing for Different Lattice Types Manufactured by FDM and FP

specimen type	lattice type	stress @ 2% strain (MPa)	ultimate strength (MPa)
FDM	unidirectional	0.058 ± 0.021	0.728 ± 0.023
	bidirectional	0.069 ± 0.006	0.342 ± 0.007
	pyramidal	0.064 ± 0.002	0.649 ± 0.014
FP	unidirectional	0.086 ± 0.027	1.280 ± 0.073
	bidirectional	0.086 ± 0.042	0.696 ± 0.096
	pyramidal	0.064 ± 0.023	0.914 ± 0.100

**Figure 8:**
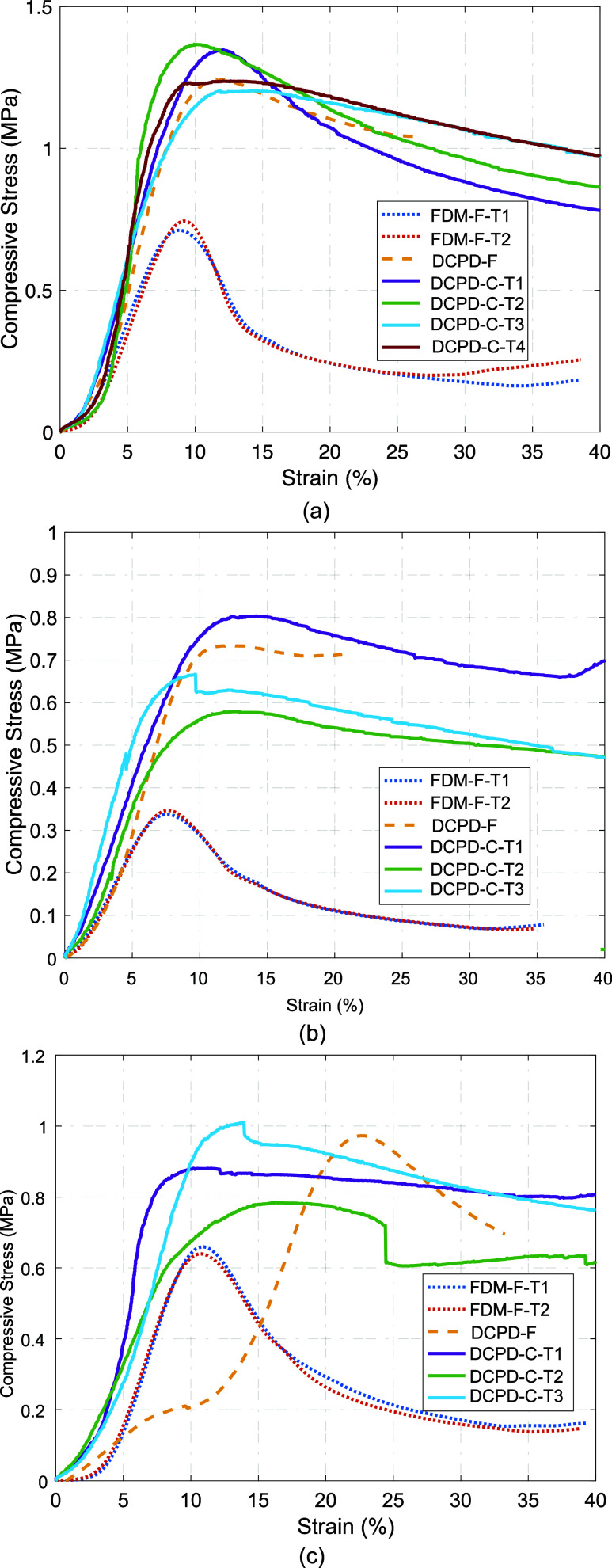
Stress–strain curves for the compressive tests for the (a) unidirectional, (b) bidirectional, and (c) pyramidal lattices.

Overall, the stress–strain response shows typical softening behavior under compression, but the FDM panels soften at a much faster rate. It should be noted that there is some abnormality in the stress–strain curve of the full pyramidal lattice DCPD panel due to warping of the panel during fabrication, resulting in stress being recorded prior to full contact between the load frame plate and the specimen and resulting in a small peak prior to the actual peak after full contact. This does not appear to affect the ultimate compressive strength of the lattice, with variation coming more from the print quality. These plots and the data in Table 2 demonstrate the improved ultimate compressive strength of the FP DCPD lattices over the FDM lattices and that cutting the specimen did not significantly alter the strength of the specimen.

Figure 9a,b show the deformed shape of the lattice during the compressive testing. In both cases, deformation can be seen in the lattice, but no lattice breakage or debonding between the lattice and the face sheet is observed. One representative failed DCPD specimen from each lattice type after the tests is shown in Figure 9c–e. The unidirectional lattice results in some lateral movement between the two face sheets, while the bidirectional and pyramidal lattices are free of the lateral movement. The primary failure mode of the lattices is deformation in the lattice struts. Only two struts showed debonding from the face sheet at the corner of the specimen, where those bonding areas were partially cut when cutting out the specimen. This also suggests that good bonding is achieved in the developed method.

**Figure 9:**
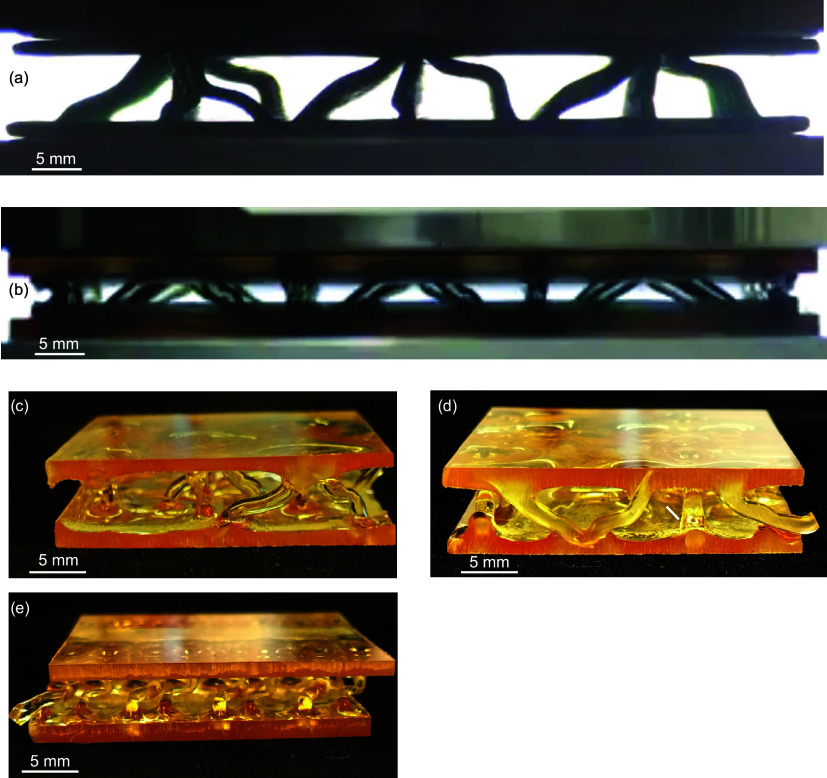
Compressive tests of the LCSPs: testing of a complete bidirectional panel with three unit cells fabricated by (a) FDM and (b) FP, where lattice deformation is observed but no lattice damage or debonding; cut DCPD specimen after tests showing the failure mode for (c) pyramidal, (d) bidirectional, and (e) unidirectional lattice types.

## 4 Summary and Conclusion

This work presents a new manufacturing method for thermoset polymer lattice core sandwich panels based on FP-based DIW. First, a lattice core is printed using FP-based DIW, considering different lattice types. The printed lattice core is then placed in an ink reservoir with a depth equal to the thickness of the desired face sheet. The setup is then moved to an oven to initiate frontal polymerization of the ink in the reservoir to quickly cure the face sheet without additional bonding processes needed. This process is repeated to form the face sheet on the other side, delivering LCSPs in a time-efficient manner. Examination of the printed parts using both a microscope and a regular camera indicates that the bonding between the face sheet and lattice core is free of voids or air bubbles and that the bonding is not the weak point under compressive loading. This method is faster than FDM, but FDM has better dimensional accuracy and consistency across the prints. The ultimate strengths of the DCPD panels are much higher than those of their FDM counterparts, and the softening after ultimate strength is much slower.

While this technique is demonstrated using small panels due to printer limitations, this technique has great potential for scalability by addressing some potential issues in a future study. First, print reliability could be improved with more sophisticated printer control algorithms and improved ink characterization before printing. Further research may also resolve the issue of panel warping during face sheet fabrication by understanding the residual deformation development in the face sheet FP process and controlling the heating process to mitigate undesired warping. Additional areas of research stemming from this may include single-process fabrication of LCSPs by using FP-based DIW only, nonplanar panel fabrication, and the introduction of fillers into the polymer matrix for composite printing.

## Supplementary Material





## Notes

A video detailing the ink preparation process. In this video, all of the steps, including the tools needed and the weight or volume measurement of each chemical, are shown to complete the ink preparation procedure. If one wants to change the ink batch size, just scale all weight and volume measures. (MP4)
A video recording of the lattice core printing process, with real-time front monitoring and printing speed adjustment. This video shows the printing process of a unidirectional core, with a regular camera view on the left and an IR camera view on the right. The black window in the top-left corner displays the printing parameters. It should be noted that the printing velocity shown there is updated in real time to ensure smooth printing. In the IR camera view, bright orange color indicates higher temperature and blue indicates lower temperature. (MP4)
